# TRIM proteins as emerging regulators of immune pathways: potential therapeutic targets in immune-related disorders

**DOI:** 10.3389/fimmu.2026.1764253

**Published:** 2026-02-11

**Authors:** Bilal Jawed, Rimsha Kanwal, Syed Khuram Zakir, Francesco Gaudio, Jessica Elisabetta Esposito, Azfar Athar Ishaqui, Stefano Martinotti, Matteo Botteghi, Elena Toniato

**Affiliations:** 1 Ente Ecclesiastico Ospedale Generale Regionale Francesco Miulli, Acquaviva delle Fonti, Italy; 2 Universita degli Studi Gabriele d’Annunzio Chieti Pescara, Chieti, Italy; 3 Universita LUM Giuseppe Degennaro Dipartimento di Medicina e Chirurgia, Casamassima, Italy; 4 Department of Clinical Pharmacy, College of Pharmacy, King Khalid University, Abha, Saudi Arabia; 5 Universita Politecnica delle Marche, Ancona, Italy

**Keywords:** adaptive immunity, autoimmune diseases, immunotherapy targets, innate immunity, pattern recognition receptors, TRIM proteins, ubiquitination

## Abstract

Tripartite motif (TRIM) proteins constitute a versatile family of E3 ubiquitin ligases that regulate key signaling pathways governing innate and adaptive immune responses. Their ability to modify receptor-proximal adaptors, transcription factors, and pattern recognition receptors positions them as central modulators of antiviral defense, cytokine production, and immune homeostasis. Dysregulated TRIM expression or activity contributes to the pathogenesis of autoimmune diseases, including SLE, Sjögren’s syndrome, rheumatoid arthritis, psoriasis, inflammatory bowel diseases, and type I diabetes. This review summarizes the role of TRIM proteins in innate and adaptive immunity and their signaling axis linked to autoimmune and immune-related pathologies. It also focuses on the emerging therapeutic potential, targets and clinical strategies for targeting TRIM proteins.

## 1 Introduction

Innate immunity functions as the initial line of defense against microbial pathogens. A diverse group of pattern recognition receptors (PRRs) present on immune cells detects a wide spectrum of pathogen-associated molecular patterns (PAMPs), including lipids, nucleic acids, and proteins (1). Among the various PRR families, Toll-like receptors (TLRs), Nod-like receptors (NLRs), and RIG-I-like receptors (RLRs) are the most extensively studied (2, 3). Activation of these receptors starts signaling cascades that involve nuclear factor κB (NF-κB) and interferon regulatory factor (IRF) families, thereby regulating the synthesis of pro-inflammatory mediators and the expression of co-stimulatory molecules, ultimately initiating adaptive immune responses (4, 5). The evidence shows that TRIM proteins interact with PRR pathways, modulating the signaling cascades triggered by PRRs to influence immune responses. These interactions suggest a regulatory function of TRIM proteins in immune signaling and the maintenance of immune homeostasis. Conversely, the adaptive immune system produces a more specialized response supported by T and B lymphocytes, which employ highly specific antigen-recognizing receptors, T cell receptors (TCRs) and B cell receptors (BCRs), accordingly. Aberrant cytokine production and dysregulated PRR signaling pathways are strongly associated with the development of autoimmune diseases (6, 7). During these pathological conditions, numerous immune cells invade significant tissues and discharge inflammatory mediators into the peripheral circulation, thereby promoting the onset and progression of autoimmune disorders (8–10).

**Figure 1:**
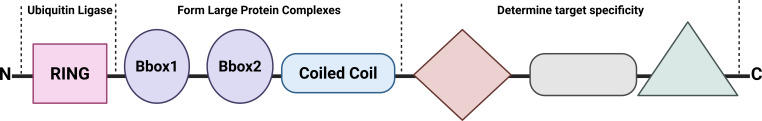
Structure domain of tripartite motif proteins.

Tripartite motif (TRIM) proteins represent a predominant family of E3 ubiquitin ligases, consisting of over 70 members in humans. These proteins feature a conserved N-terminal tripartite “RBCC” motif, comprising a RING finger domain, one or two B-box domains, and a coiled-coil domain (**Figure 1**) (11). Ubiquitination represents a vital post-translational modification mechanism in eukaryotic cells, regulating vital biological processes including DNA damage repair, cell cycle progression, and signal transduction (12). The proliferation of the TRIM multigene family closely aligns with the evolution of innate and adaptive immunity, suggesting that TRIM proteins are crucial in modulating complex immune functions (13).

Accumulating evidence indicates that the expression of TRIM proteins is significantly influenced by diverse stimuli, including interferons (IFNs), lipopolysaccharides (LPS), and viral components (14, 15). Recent studies further highlight the significance of TRIM proteins in regulating host immune responses and the pathophysiology of autoimmune diseases (16). TRIMs have demonstrated an impact on key immunological processes, including the production of type I interferons (IFN-1) and pro-inflammatory cytokines, such as interleukin-1β (IL-1β) (17, 18). A defining feature of TRIM proteins is their context-dependent capacity to function as either positive or negative regulators of immune signaling, depending on cellular state, stimulus and temporal dynamics. In this review, we comprehensively evaluate the immunoregulatory roles of TRIM family proteins and their mechanistic involvement in modulating innate and adaptive immune signaling pathways. Furthermore, we examine current evidence linking dysregulated TRIM activity to autoimmune and immune-mediated disorders, and discuss the therapeutic potential of targeting TRIM proteins, along with possible targeting strategies for future clinical interventions.

## 2 Structural organization of TRIM proteins

TRIM proteins are structurally defined by a conserved N-terminal RBCC motif, comprising a RING finger domain, BBox domains, and a CC region, followed by a varied C-terminal domain (**Figure 1**) (19). This modular arrangement constitutes the structural foundation for their diverse biological functions. These roles encompass immunological regulation, apoptosis, and transcriptional control.

The Really Interesting New Gene (RING) finger domain, generally composed of 40-60 amino acid residues (20), serves as the catalytic core of TRIM proteins. As a zinc-binding motif, it enables the transfer of ubiquitin from E2 conjugating enzymes to designated substrate proteins, thus establishing E3 ubiquitin ligase activity. The RING domain structurally aligns two zinc ions in a crossed-brace conformation, which is essential for maintaining its enzymatic function (21). Adjacent to the RING domain are one or two B-box domains, each consisting of approximately 32-42 amino acid residues (20). These domains are zinc-binding motifs that facilitate protein-protein interactions, mediating the homo- or hetero-oligomerization of TRIM proteins. While the exact molecular mechanisms of B-box areas remain less well understood compared to the RING domain, mutations within B-box regions have been associated with autoimmune pathological diseases, underscoring their functional relevance (22). The B-box domains are also thought to affect the stability and oligomerization of TRIM proteins, therefore influencing their structural integrity and regulatory potential (23).

**Figure 2:**
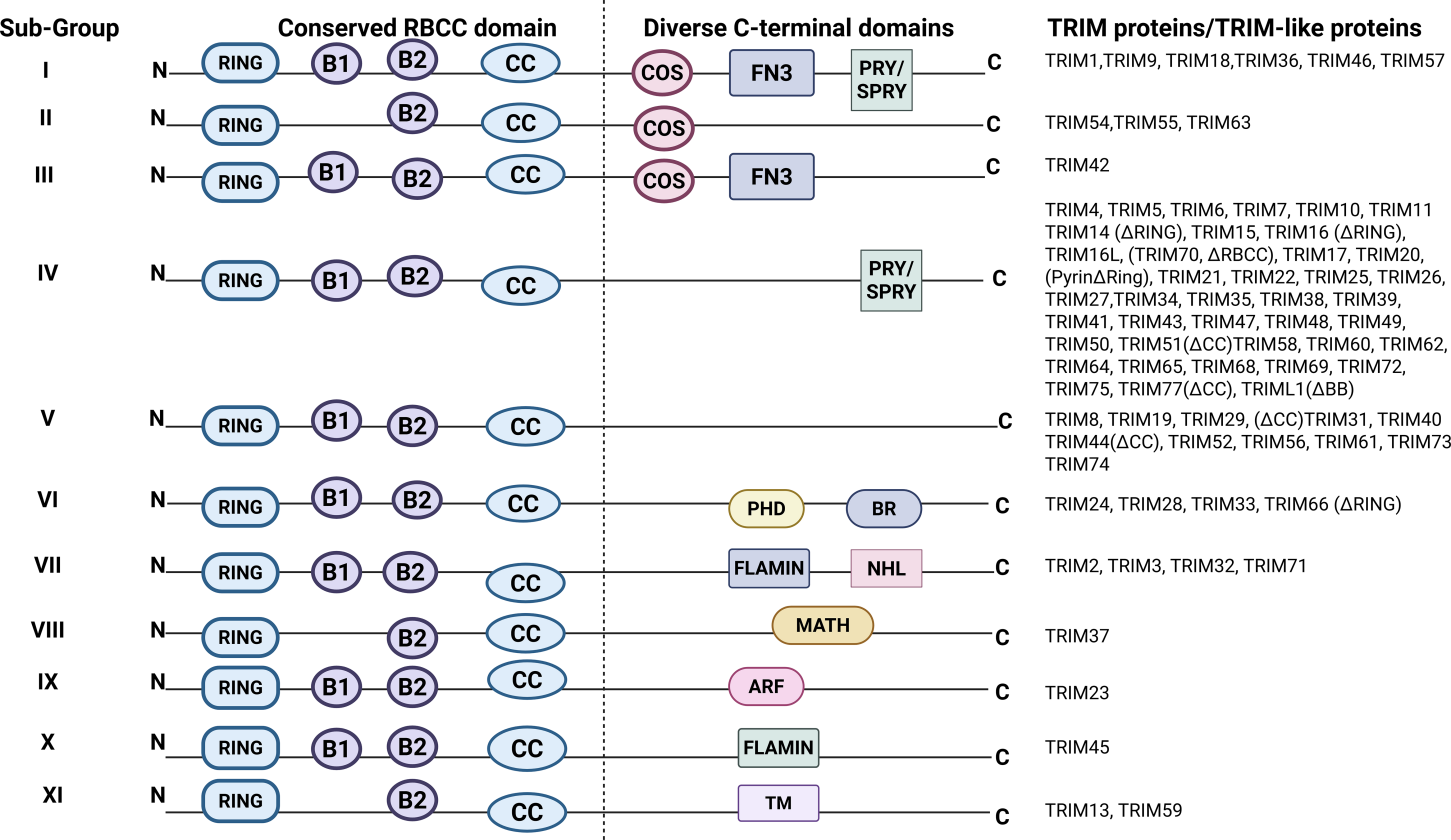
Structural classification of TRIM/RBCC proteins: The conserved N-terminal RBCC motif composed of the RING, B-box 1, B-box 2, and coiled-coil domains. Their structurally diverse C-terminal regions define 12 TRIM subfamilies and incorporate modules such as COS (COS box motif), FN3 (Fibronectin type III motif), PRY, SPRY (SPla and the RYanodine receptor), PHD (Plant homeodomain), BR (bromo domain), FILAMIN (filamin-type immunoglobulin), NHL (NCL 1/HT2A/LIN-41), MATH (meprin and TRAF homology domain), ARF (ADP ribosylation factor)/SAR, TM (transmembrane motif), and a variable domain.

The coiled-coil region serves as a crucial region for the oligomerization of TRIM proteins, facilitating the formation of higher-order complexes. This domain promotes the assembly of functional complexes, which enhance the concentration of enzymatic components and offer a scaffolding framework for interacting partners. Consequently, the coiled-coil region is vital for determining the specificity, efficiency, and amplification of intracellular signaling cascades. Following the coiled-coil region, the C-terminal domain of TRIM proteins has considerable structural variability, which dictates their functional diversity. TRIM proteins are categorized into 11 subfamilies (C-I to C-XI) based on their composition of this particular region. The most predominant C-terminal module is the SPRY (also known as B30.2) domain, although other domains such as PRY/SPRY, MATH, PHD, and NHL are also represented (22). The PRY/SPRY domain is a key determinant of innate immune function in several TRIM proteins. It supports viral protein recognition and subcellular targeting (24). This domain in TRIM25 mediates RNA binding and is essential for its ubiquitination activity. TRIM23 modulates innate immune signaling through its ARF-like C-terminal domain, which possesses intrinsic GTPase activity. This domain supports TRIM23-mediated, K27-linked ubiquitination of TBK1 and promotes type I IFN responses. Another common C-terminal domain is the COS domain, which mediates association with microtubules and contributes to antiviral activity. The FN3 domain supports interactions with DNA and heparin. The PHD domain is predominantly found in nuclear TRIM proteins and has been linked to chromatin-associated transcriptional control. These domains mediate selective substrate recognition, subcellular localization, and pathway activation, thus providing the structural basis for the remarkable functional diversity observed among members of the TRIM family. This heterogeneity allows TRIM proteins to engage in a wide range of molecular interactions, thereby influencing multiple signaling pathways and expanding their functional versatility (**Figure 2**) (22, 25). Among the extensive TRIM family, many members have been characterized for their crucial involvement in immune regulation, particularly within innate immune responses to viral infections.

## 3 Mechanistic framework and functional diversity of TRIM proteins in immune regulation

Mechanistically, many TRIM proteins function as E3 ubiquitin ligases, facilitating the transfer of ubiquitin molecules to specific substrates, thus modulating their stability, activity, and trafficking in immunological signaling (21). TRIM-mediated ubiquitination includes both canonical K48-linked polyubiquitin chains that direct substrates for proteasomal degradation, and non-degradative linkages like K63 and K27-linked chains promote signal propagation through scaffold formation and adaptor recruitment (26). Through these ubiquitination-dependent and ubiquitin-like modifications, TRIM proteins integrate structural features with ubiquitin linkage diversity, regulate signaling hubs and multiprotein complexes across innate and adaptive immunity (**Table 1**) (71).

**Table 1:** TRIM proteins mediated regulatory functions in immune responses.

TRIM protein	Target molecule	Biochemical action	Effect on signaling	Refs.
TRIM4	RIG-I (CARDs)	Mediates K63-linked polyubiquitination of RIG-I CARDs to enhance receptor activation.	Positive regulator that enhances IRF3 & NF-κB activation and promotes IFN-β transcription.	(27)
TRIM5α	TAK1 (via free/unanchored K63 ubiquitin chains)	Generates free K63-linked polyubiquitin chains that acts as scaffolds to recruit & activate the TAK1 complex.	Positive regulator that promotes TAK1 activation & downstream AP-1/NF-κB signaling.	(28, 29)
TRIM9	β-TrCP	Interacts with β-TrCP and stabilizes IκBα and p100	Negative regulator of canonical and non-canonical NF-κB pathways through stabilization of inhibitory components.	(30)
TRIM9s	TBK1	Undergoes self-K63-linked autoubiquitination recruiting GSK3β to TBK1 & facilitating TBK1 phosphorylation.	Positive modulator of TBK1 activation and downstream IRF3 signaling.	(31)
TRIM11	TBK1	Physically associates with TBK1 and inhibits its phosphorylation and activation.	Negative regulator that dampens TBK1-mediated IFN induction.	(32)
TRIM12c (mouse)	TRAF6	Interacts with TRAF6 and induces TRAF6 ubiquitination.	Positive regulator in mouse models; stimulates IFN & NF-κB pathways (Trim12c is a Trim5 family member).	(33)
TRIM13	TRAF6	Induces TRAF6 ubiquitination; specific ubiquitin linkage type was defined in follow-up studies.	Positive modulator of TLR2-mediated NF-κB activation; enhances cytokine production.	(34)
TRIM13	MDA5	Interacts with MDA5 & attenuates its downstream signaling; precise ubiquitin linkage not detailed.	Negative regulator of MDA5-mediated IFN-I production.	(35)
TRIM14	MAVS	Serves as a scaffold linking MAVS and NEMO to assemble the signaling complex; lacks catalytic RING domain.	Positive modulator that strengthens MAVS-mediated NF-κB and IRF3 activation.	(36)
TRIM14	cGAS	Recruits deubiquitinase USP15 to prevent K48-linked degradation of cGAS.	Positive regulator that stabilizes cGAS and promotes cGAS-mediated IFN responses.	(37)
TRIM19 IV (PML-IV)	Pin1 (and IRF3 interactions)	Regulates the cellular distribution of Pin1 and associates with activated IRF3 in a context-dependent manner.	Modulatory function influencing IRF3 stability & localization, thereby adjusting IFN output.	(38, 39)
TRIM19 (PML)	RelA/p65	Physical interacts with RelA/p65 & modulates transcriptional activity.	Regulatory function limiting NF-κB transcriptional output by preventing RelA/p65 binding to target genes.	(40)
TRIM20 (Pyrin/MEFV)	IκBα and p65 (NF-κB axis)	PYD domain interacts with IκBα and p65 to promote IκBα degradation & p65 nuclear translocation; functionally linked to inflammasome activity.	Context-dependent regulator that can enhance NF-κB activation and is associated with autoinflammatory disease (FMF) when mutated.	(41)
TRIM21 (Ro52/SS-A)	IRF7/IRF3	Ubiquitinates IRF7 and IRF3 causing proteasomal degradation; also mediates autophagic degradation of IRF3.	Negative feedback regulator of IFN-I production; prevents excessive antiviral signaling.	(42, 43)
TRIM21	TAK1	Generates free K63-linked polyubiquitin chains that activate TAK1.	Context-dependent positive regulator promoting TAK1 activation and downstream NF-κB & MAPK signaling.	(44)
TRIM21	IKKβ	Catalyzes monoubiquitination of IKKβ which targets IKKβ to autophagolysosome for degradation.	Negative regulator of NF-κB signaling through reduction of IKKβ levels.	(45)
TRIM21	DDX41	Catalyzes K48-linked polyubiquitination and proteasomal degradation of DDX41.	Negative regulator that suppresses DDX41-dependent DNA-sensing & IFN-I production.	(46)
TRIM22	TAB2	Targets TAB2 for degradation, thereby limiting TRAF6/TAB2-dependent signaling.	Negative regulator of NF-κB activation.	(47)
TRIM23	NEMO; TRAF6	Mediates K27-linked ubiquitination of NEMO & promotes TRAF6 autoubiquitination	Positive modulator of NF-κB and IFN signaling supporting TLR3 & RLR responses.	(48, 49)
TRIM25	RIG-I (CARDs)/MAVS	Catalyzes K63-linked polyubiquitination of RIG-I CARDs (notably K172)leading to activation of RIG-I; also ubiquitinates MAVS to facilitate signal release.	Positive regulator that promotes RIG-I -MAVS interaction and induces robust IFN-I production.	(50, 51)
TRIM26	NEMO/IRF3	Bridges TBK1 to NEMO (positive) but also promotes K48-linked ubiquitination & degradation of IRF3 (negative).	Context-dependent regulator that can both promote and suppress TBK1-IRF3 axis depending on conditions.	(52, 53)
TRIM27 (RFP)	IKKs (IKKα/IKKβ/IKKϵ)	Physically interacts with canonical & noncanonical IKK family members.	Generally negative regulator of IFN and NF-κB signaling; silencing enhances ISRE & NF-κB activity.	(54)
TRIM28 (KAP1)	IRF7	Promotes SUMOylation of IRF7 (E3-like activity in the modification process).	Negative regulator of IRF7 transactivation resulting in suppression of IFN transcription.	(55)
TRIM29	NEMO	Mediates K48-linked polyubiquitination of NEMO leading to proteasomal degradation.	Negative regulator of NF-κB and antiviral responses; suppresses IFN & cytokine production.	(56)
TRIM30α (mouse)	TAB2/TAB3 (TAK1 complex)	Interacts with TAK1 complex and promotes TAB2 & TAB3 degradation through a non-proteasomal mechanism.	Negative regulator of LPS-induced NF-κB signaling by limiting TAK1 activation.	(57)
TRIM30α (mouse)	STING	Promotes K48-linked ubiquitination and proteasomal degradation of STING.	Negative regulator that attenuates STING-mediated type I IFN induction.	(58)
TRIM31	MAVS	Mediates K63-linked polyubiquitination at MAVS Lys 10, 311, and 461 driving prion-like MAVS aggregation.	Positive regulator that promotes MAVS aggregation and amplifies signaling.	(59)
TRIM32	STING	Induces K63-linked polyubiquitination of STING promoting TBK1 association.	Positive regulator that strengthens STING signaling.	(60)
TRIM38	TRIF	Catalizes K48-linked polyubiquitination of TRIF (targets TRIF K228)leading to proteasomal degradation.	Negative regulator of TLR3/TLR4 TRIF-dependent IFN-I and inflammatory responses; limits cytokine & IFN production.	(61)
TRIM38	TRAF6	Induces K48-linked ubiquitination of TRAF6 resulting in proteasomal degradation.	Negative regulator of MyD88-dependent NF-κB activation; attenuates proinflammatory signaling.	(62)
TRIM38	cGAS/STING	Mediates SUMOylation of cGAS and STING early during infection, protecting them from degradation; SUMO is removed later to terminate signaling.	Temporal positive regulator that stabilizes cGAS & STING in early infection stages and limits them later.	(63)
TRIM40	NEMO	Mediates neddylation of NEMO (modification with NEDD8) that alters NEMO function.	Negative modulator of NF-κB signaling; suppresses activation via altered NEMO modification.	(64)
TRIM44	MAVS	Binds MAVS & prevents its ubiquitination and degradation, stabilizing the adaptor.	Positive regulator that stabilizes MAVS and enhances antiviral signaling.	(65)
TRIM56	TRIF (TLR3)	Physically interacts & forms a scaffold with TRIF; E3 activity is dispensable for TRIF complex formation.	Positive regulator that promotes TLR3 signaling and type I IFN production.	(66)
TRIM56	STING	Catalyzes K63-linked polyubiquitination of STING to enhance TBK1 recruitment.	Positive regulator and amplifies STING-dependent IFN-I responses.	(67, 68)
TRIM65	MDA5 (helicase domain)	Catalyzes K63-linked polyubiquitination of MDA5 (K743)promoting MDA5 oligomerization and activation.	Positive regulator which facilitates MDA5-driven antiviral IFN-I responses.	(69)
TRIM68	TFG	Targets TFG for lysosomal degradation, reducing availability of adaptor proteins for IFN signaling.	Negative regulator which suppresses IFN-β production.	(70)

Pattern recognition receptors (PRRs) expressed by innate immune cells act as key sensors of molecular patterns associated with pathogens (PAMPs) derived from microbes and damage-associated molecular patterns (DAMPs) released during cellular stress or tissue damage (72). Through this detection function, PRRs initiate innate immune responses. Numerous TRIM proteins regulate innate immune signaling by modulating the activity of essential PRRs, including Toll-like receptors (TLRs), RIG-I-like receptors (RLRs), C-type lectin receptors (CLRs), NOD-like receptors (NLRs), and cytosolic DNA sensors (CDSs). Ligand engagement of these PRRs triggers their activation, which involves the recruitment of adaptor proteins and the initiation of downstream signaling cascades involving transcription factors such as NF-κB and IRF. These pathways lead to the production of type I IFNs, chemokines, and pro-inflammatory cytokines (73). These signaling pathways enable rapid transcriptional responses that are essential for pathogen control and coordination of adaptive immunity. TRIM proteins tune these pathways at multiple nodes. For example, TRIM25 promotes RIG-I ubiquitination through its SPRY domain-mediated substrate recognition, which mediates interaction with the RIG-I CARD domains, enabling K63-linked ubiquitination via its RING domain. This facilitates its activation and amplifies antiviral responses, thereby improving RIG-I-mediated antiviral signaling (74). In contrast, TRIM8 negatively regulates TLR3/TLR4 signaling by PRY/SPRY domain-dependent targeting with the adaptor protein TRIF. This interaction promotes RING-dependent polyubiquitination of TRIF and disrupts formation of the TRIF-TBK1 signaling complex. As a result, downstream type I interferon and inflammatory signaling are reduced (75). Beyond innate immunity, TRIM proteins also influence adaptive immunological responses by influencing T cell development and activation, along with B cell differentiation and antibody synthesis (73).

The functional scope of TRIM proteins further extends to the regulation of autophagy, apoptosis, and cell cycle progression. Structural features provide the basis for this functional diversity. The conserved RBCC core confers E3 ubiquitin ligase activity while variable C-terminal domains determine substrate specificity and pathway selection (21). SPRY domains mediate selective substrate recognition and enable TRIM proteins such as TRIM25 and TRIM21 to engage immune sensors and adaptors (74, 76). TRIM21 displays functional specialization through its C-terminal PRY/SPRY domain, which mediates high-affinity binding to the Fc region of antibodies associated with intracellular pathogens. This mode of substrate recognition enables TRIM21 to function as a cytosolic antibody receptor. It couples pathogen sensing to RING-mediated ubiquitination and the activation of innate immune signaling (76). The immunomodulatory and antiviral functions of TRIM32 are mediated through its C-terminal NHL repeat domain. This domain supports selective interactions with viral proteins and host RNA-associated factors. Such substrate recognition links RING-dependent ubiquitination to post-transcriptional regulation of immune responses and promotes antiviral restriction and immune homeostasis (22). ARF-domains possess GTPase activity and direct K27-linked ubiquitination of TBK1, as demonstrated by TRIM23, which promotes antiviral signaling (22, 76). Additionally, TRIM proteins are integral to the regulation of inflammasome activation, underscoring their essential role in maintaining immune homeostasis and coordinating responses to cellular stress (76).

Ubiquitin linkage diversity further encodes regulatory outcomes. The ability of TRIM proteins to modulate multiple signaling pathways establishes them as central regulators of immune function. They are essential in both the activation and control of inflammatory responses, as well as in maintaining immunological tolerance. K63 chains amplify signaling through scaffold formation while K48 chains terminate responses by proteasomal degradation (26, 71). TRIM38 illustrates temporal control by stabilizing cGAS STING via SUMOylation early and later promoting K48-linked degradation to resolve signaling (77). Similar context-dependence is observed for TRIM21, which functions as a cytosolic Fc receptor to activate innate signaling yet limits IFN responses through targeted degradation of IRF proteins (76). These examples establish context-dependent regulation as a defining feature of TRIM biology. Dysregulation of TRIM protein activity or ubiquitination-proteasome system has been involved in various immune-mediated conditions, such as systemic lupus erythematosus, rheumatoid arthritis, and multiple sclerosis, emphasizing their potential as promising therapeutic targets (78).

The combination of structural complexity, ubiquitin linkage diversity, and context-dependent regulation enables TRIM proteins to act as either positive or negative regulators of immune signaling. This functional plasticity enables immune homeostasis but also represents a major challenge for therapeutic targeting because indiscriminate modulation may disrupt the balance between protective immunity and immunopathology. These aspects are further explored in subsequent sections of this review.

## 4 TRIM proteins in innate immune regulation

### 4.1 TRIM-mediated regulation of TLR pathways in immune response

**Figure 3:**
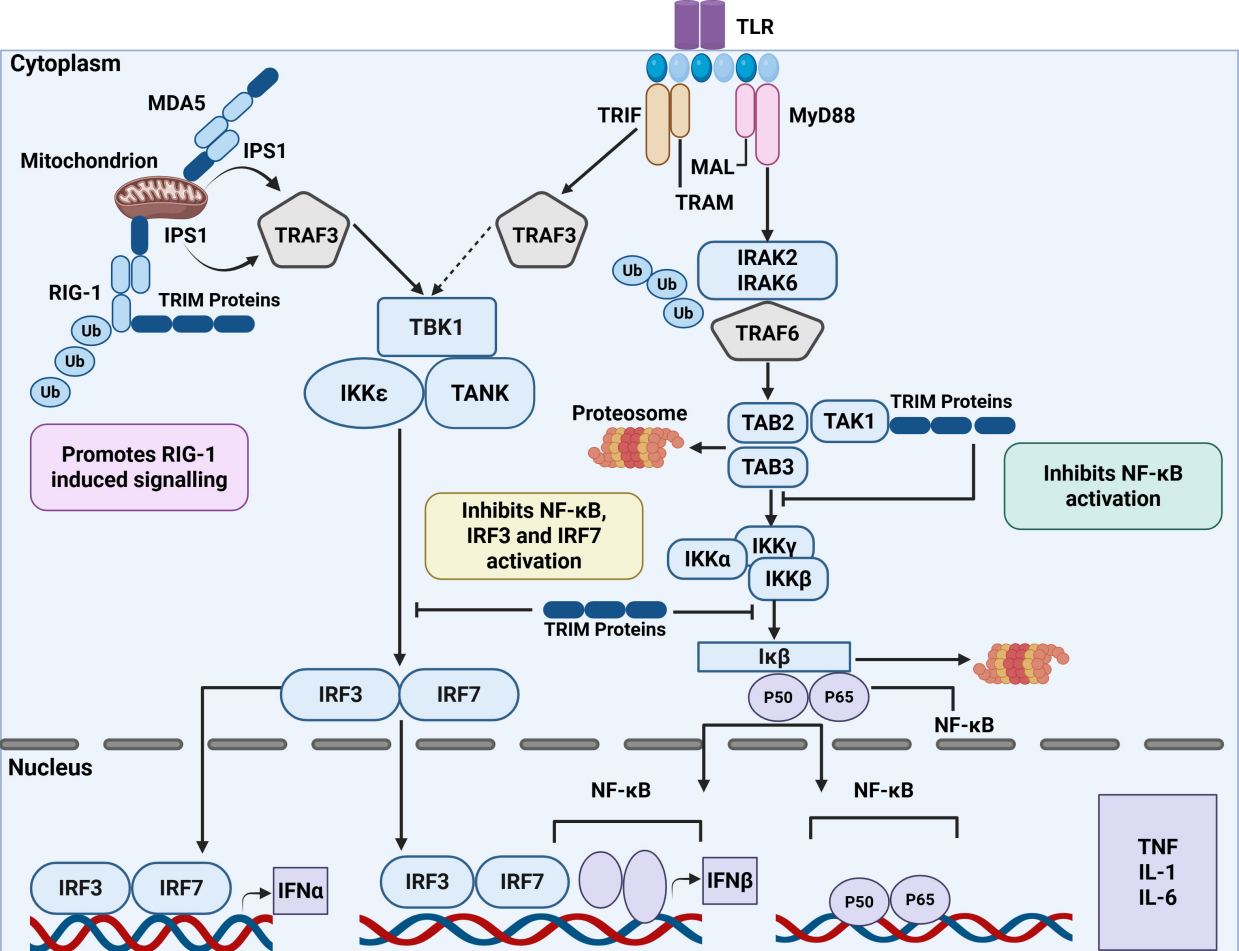
Schematic diagram of TRIM proteins-mediated regulation of innate immune signaling pathways. TLRs, Toll-like receptors; NF-κB, nuclear factor-κB; IFNs, interferon; IRF3, interferon-regulatory factor 3; IRF7, interferon-regulatory factor 7; IL-1, -1; IL-6, interleukin-6; RIG-I, retinoic-acid-inducible gene I; MDA5, melanoma differentiation-associated gene 5; TAK1, transforming growth factor-β (TGFβ)-activated kinase 1; IRAK, IL-1 receptor-associated kinase; MyD88, myeloid differentiation gene response 88; IPS1, IFNB-promoter stimulator 1; IκB, inhibitor of NF-κB; MAL, MyD88-adaptor-like protein; IKKi, IκB kinase inducible gene; TAB2, TAK1-binding protein 2; TAB3, TAK1-binding protein 3; IKKs, IκB kinase; TRAF, TNF-receptor-associated factor; TANK, -family member-associated NF-κB activator -binding kinase-1; TRIF, Toll/IL-1-receptor domain-containing adaptor protein inducing IFNβ; TRAM, -related adaptor molecule; TBK1, TANK-binding kinase 1; TRIM, tripartite motif protein; Ub, ubiquitin.

The Toll-like receptor (TLR) family comprises of more than ten members, among which TLR2 and TLR4 have been most extensively investigated. Members of the TLR family are expressed by a wide range of immune cell types, including dendritic cells (DCs), macrophages, B cells and other innate and adaptive immune cells. TLRs recognize extracellular and endosomal PAMPs, initiating signaling through Toll/IL-1 receptor (TIR) domain-containing adaptor proteins (79). These receptors activate two primary adaptor-dependent signaling cascades: the myeloid differentiation gene response 88 (MyD88)-dependent pathway, utilized by all TLRs except TLR3, and the TIR-domain-containing adapter-inducing interferon-β (TRIF)-dependent pathway, employed by TLR3 and, through the TRIF-related adaptor molecule (TRAM), by TLR4 (80) (**Figure 3**). TRIM proteins regulate both the MyD88 and TRIF-dependent signaling branches at many stages, ranging from receptor-proximal adaptor molecules to downstream kinases and transcriptional regulators. They exert TLR signaling outputs through both ubiquitin-dependent and ubiquitin-independent mechanisms, either potentiate or suppress immune activation. These signaling cascades ultimately govern the expression of inflammatory cytokines, chemokines, and type I interferons (IFNs). Understanding the regulatory involvement of TRIM proteins in these pathways is therefore crucial for clarifying the molecular mechanisms that link TLR signaling to trained immunity and immune homeostasis. TLR ligands have been shown to induce long-term functional reprogramming of innate immune cells through metabolic and epigenetic mechanism (81).

#### 4.1.1 TRIF signaling in TLR pathways and regulation by TRIM proteins

After TLR4 is endocytosed, the adaptor protein TRAM (TICAM-2) translocates to the endosomal compartment. This aligns with the TIRAP-MyD88 complex dissociating from the plasma membrane (82). Within the endosome, TLR3 and internalized TLR4 bind the TRAM-TRIF complex, which forms a core scaffold for signal relay. TRIF works with different TNF receptor-associated factors (TRAFs) to trigger two main signaling pathways (83). In one pathway, TRIF associates with TRAF6, leading to the recruitment and activation of the TGF-β-activated kinase 1 (TAK1) complex. Activated TAK1 subsequently stimulates the IκB (inhibitor of NF-κB)-kinase (IKK) complex, which comprises the NF-κB essential modulator (NEMO/IKKγ) and the mitogen-activated protein kinase (MAPK) pathways (84). TNF-α, IL-6 and IL-12 are pro-inflammatory cytokines that are produced when these cascades result in the nuclear translocation of the transcription factors NF-κB and activator protein-1 (AP-1) (85). In the alternative branch, TRIF binds TRAF3, which in turn activates the IκB kinase inducible gene (IKKi) and TANK-binding kinase 1 (TBK1). This signaling axis promotes the phosphorylation and dimerization of interferon regulatory proteins IRF3 and IRF7, which subsequently translocate to the nucleus to initiate transcription of type I interferons (IFN-α and IFN-β), essential for antiviral defense (86).

Several TRIM proteins have been identified as essential modulators of TRIF-dependent signaling. TRIM8 exhibits context-dependent regulatory function in innate signaling. Through PRY/SPRY domain-mediated interaction, this pathway is negatively regulated by disrupting the TRIF-TBK1 interaction, thus reducing IRF3 activation and limiting type I IFN production. In contrast, TRIM8 can also enhance K63-linked polyubiquitination of TAK1 and promote NF-κB activation (87). Similarly, TRIM32 functions as a negative regulator of TLR3/4 mediated signaling by enhancing the binding of TRIF to the adaptor TAX1BP1, which facilitates its selective autophagic degradation and attenuates immune responses (88). TRIM38 mediates the ubiquitin-dependent degradation of TRIF and other adaptor molecules like TRAF6 and NAP1, acting as an inducible inhibitory regulator of TLR signaling. Through K48-linked ubiquitination, TRIM38 targets these adaptors for proteasomal or lysosomal degradation, therefore downregulating the IRF3/TBK1 and NF-κB pathways, a classical feedback mechanism that prevents excessive cytokine or IFN production (61). Conversely, certain TRIMs enhance TRIF-dependent antiviral responses. TRIM56, an E3 ligase, augments TLR3-mediated IFN production by interacting with TRIF and promoting the activation of downstream signaling components (66). TRIM14, though lacking a conventional RING domain, serves as a scaffold that stabilizes nucleic acid sensors and facilitates TBK1 recruitment, thereby supporting TRIF-linked antiviral signaling in both DNA and RNA sensing contexts (89). The regulatory impact of TRIM proteins on TRIF signaling is highly context-dependent. Early positive regulators, such as TRIM56 and TRIM14 enhance type I IFN production to promote effective pathogen elimination, whereas inducible negative regulators on TRIF signaling like TRIM38 and TRIM8 act at later stages to terminate signaling and prevent immunopathology. Disruption in this context-dependent balance mechanism can lead to uncontrolled inflammation, contributing to the development of autoimmune and inflammatory diseases in both experimental and clinical studies.

#### 4.1.2 MyD88-mediated TLR pathways and regulation by TRIM proteins

The majority of TLRs, including TLR2, TLR5, TLR7, TLR8 and TLR9, activate the MyD88-dependent signaling cascade as their primary pathway. This pathway mediates the rapid induction of pro-inflammatory cytokines that are critical for early immune defense (90). The adaptor protein MyD88 is drawn to the TIR domain of TLRs upon ligand recognition at the plasma membrane, starting the formation of the Myddosome, a higher-order signaling complex. This complex consists of MyD88 and interleukin-1 receptor-associated kinases (IRAK1 and IRAK4), which are subsequently activated and interact with TRAF6, a key E3 ubiquitin ligase. TRAF6 catalyzes K63-linked polyubiquitination processes that attract and activate the TAK1- TAB complex. Activated TAK1 subsequently triggers the MAPK pathways and the IκB kinase (IKK) complex, which includes IKKα, IKKβ, and NEMO (IKKγ). These signaling cascades result in the nuclear translocation of transcription factors NF-κB and AP-1, which promote the transcription of inflammatory substances, including TNF-α, IL-1β, IL-6, and other chemokines essential for host defense (85, 91, 92).

Several TRIM proteins have been shown to regulate MyD88-mediated signaling, either by enhancing or restraining specific steps in the cascade. TRIM13 catalyzes K29-linked polyubiquitination of TRAF6, which positively promotes downstream signaling and NF-κB activation in TLR2-associated immunological responses (62). Conversely, TRIM38 act as a negative regulator by promoting TRAF6 to be ubiquitinated by K48, which results in its proteasomal breakdown and in turn, the suppression of NF-κB activation in macrophages (62). Similarly, TRIM32 suppresses TLR-mediated signaling through the targeted degradation of key signaling intermediates, contributing to the negative feedback control of inflammatory responses (93). These regulatory loops collectively act to fine-tune cytokine production and prevent excessive tissue-damaging inflammation. Collectively, these demonstrate that distinct TRIM proteins selectively modify the shared adaptor protein through different ubiquitin linkages, thereby exerting opposing regulatory effects on MyD88-dependent TLR signaling and NF-κB activation (see **Table 1**).

In contrast, certain TRIMs act as positive modulators. TRIM5 enhances MyD88-dependent signaling by generating free K63-linked polyubiquitin chains via its E3 ubiquitin ligase function. In cooperation with the E2 conjugating enzymes UEV1A and UBC13, TRIM5 scaffolds the recruitment of the TAK1 complex, resulting in the activation of TAK1 and subsequent transcription factors, including AP-1 and NF-κB (94). Similarly, TRIM8 positively promotes K63-linked polyubiquitination of TAK1, thereby amplifying NF-κB activation triggered by TNFα- and IL-1β under certain stimulatory conditions (95). Collectively, these findings illustrate that TRIM proteins serve as both activators and repressors within MyD88-dependent TLR signaling, providing a dynamic regulatory balance that ensures effective immune defense while preventing excessive autoimmunity responses.

### 4.2 TRIM proteins and RLR signaling pathways in immune response

The three main members of the RIG-I-like receptor (RLR) family are laboratory of genetics and physiology 2 (LGP2), retinoic acid-inducible gene I (RIG-I), and melanoma differentiation-associated protein 5 (MDA5) (96). Except for LGP2, RLRs have two N-terminal caspase recruitment domains (CARDs), a C-terminal regulatory domain (RD) and a central DExD/H-box helicase domain. RLRs identify viral RNA molecules by their unique DExD/H-box RNA helicase, thereby triggering antiviral immune responses. Upon recognition and binding of PAMPs, RIG-I and MDA5 change their conformation, exposing their CARD domains and allowing them to engage with the mitochondrial antiviral signaling protein (MAVS). This interaction subsequently triggers downstream activation of the NF-κB and IFN pathway of signaling (97, 98). Although LGP2 lacks CARD domains necessary for direct signaling, it is a positive regulator of antiviral responses mediated by RIG-I and MDA5. LPG2 enhances the sensitivity of RIG-I and MDA5 to viral infection by facilitating viral RNA recognition through its ATPase domain. Both RIG-I and MDA5 are extremely regulated by post-translational changes, ensuring their rapid activation upon viral detection while preventing excessive immune responses (99, 100).

Recent studies have established that TRIM proteins are essential in modulating RLR-dependent antiviral signaling. TRIM25, one of the most well-characterized E3 ubiquitin ligases in this context, promotes RIG-I oligomerization and its interaction with MAVS, leading to efficient downstream signal transduction (50). The ubiquitin-specific peptidase 15 (USP15) enhances TRIM25 stability by inhibiting its K48-associated ubiquitination, thus positively modulating the TRIM25-RIG-I signaling axis (51). Structural analyses of TRIM25 have identified a potential binding pocket within its SPRY domain that mediates interaction with RIG-I. This discovery suggests that small-molecule compounds targeting this domain could serve as therapeutic agents to inhibit aberrant RIG-I activation in autoimmune or hypersensitivity disorders (101).

Other TRIM proteins also contribute to the regulation of RLR signaling. TRIM35 acts as a positive regulator by catalyzing K63-dependent polyubiquitination of TRAF3, which leads to the formation of the TRAF3-MAVS-TBK1 signaling complex and facilitates activation of IRF3/7 (102). Similarly, TRIM14, a mitochondria-associated adaptor, recruits the IKKγ/NEMO adaptor to MAVS, thereby enhancing RIG-I-dependent activation of IRF3 and NF-κB pathways (36). In addition, several other TRIM family members, including TRIM4 (27), TRIM65 (69), TRIM31 (59), TRIM13 (103), and TRIM44 (65), have been reported to regulate RLR signaling through direct ubiquitination of RLRs or MAVS. Depending on the cellular context and target lysine residues, these TRIMs can catalyze activating (K63-linked) or inhibitory (K48-linked) ubiquitin chains. Some also control MAVS turnover or aggregate clearance, influencing the duration and magnitude of interferon responses (see **Table 1**). Dysregulated TRIM-RLR interactions can modify host susceptibility to RNA viruses and lead to chronic interferonopathies when negative feedback mechanisms fail. Given that RLR signaling is central to antiviral defense, TRIM proteins that potentiate this pathway represent promising candidates for antiviral enhancement, whereas those that suppress it may serve as potential therapeutic targets in interferon-mediated autoimmune diseases.

### 4.3 TRIM proteins in the cGAS-STING pathway in immune response

Cyclic GMP-AMP synthase (cGAS), an enzyme that is triggered by double-stranded DNA and acts as a pattern recognition receptor for aberrant or pathogen-derived DNA in the cytoplasm, is the main detector of cytosolic DNA (104). A conformational shift in cGAS upon attachment to double-stranded DNA catalyzes the synthesis of cyclic GMP-AMP (cGAMP), a secondary messenger. cGAMP subsequently interacts with the stimulator of interferon genes (STING) on the endoplasmic reticulum membrane, triggering STING oligomerization and its translocation to the Golgi apparatus. Activated STING provides a platform for the recruitment of kinases TBK1 and IKKi, which phosphorylate the interferon regulatory factors IRF3 and IRF7. Phosphorylated IRF3/7 dimerize and translocate to the nucleus, initiating transcription of IFN-α/β and interferon-stimulated genes (ISGs), thereby establishing an antiviral state. In parallel, STING activation induces NF-κB-dependent transcription, promoting the production of pro-inflammatory cytokines and coordinating a comprehensive innate immune response (105, 106).

TRIM family proteins serve as critical modulators of the cGAS–STING pathway, providing either positive or negative regulation to maintain immune balance. TRIM56 enhances cGAS activity through monoubiquitination, increasing cGAMP production and promoting downstream STING activation. Both TRIM56 and TRIM32 have been reported to promote K63-linked polyubiquitination of STING in response to cytosolic DNA stimulation, facilitating antiviral signaling. However, more recent studies suggest that instead of directly ubiquitinating STING, these TRIMs may generate unanchored polyubiquitin chains that activate NEMO, which, in cooperation with IKKβ and TBK1, drives IRF3 and NF-κB activation (60, 67, 68). Conversely, TRIM30α (58) and TRIM29 (56) mediate K48-dependent ubiquitination of STING, leading to its proteasomal destruction. The negative regulation of STING-dependent signaling in macrophages and DCs during herpes simplex virus type 1 (HSV-1) infection attenuates the antiviral response. TRIM14 stabilizes cGAS by preventing its K48-linked polyubiquitination and recruiting deubiquitinases such as USP14, thereby sustaining type I IFN signaling and enhancing antiviral innate immunity (37). Similarly, TRIM38 also displays context-dependent regulatory role and negatively regulates TLR and RIG-I-like receptor pathways (61). Under basal conditions, TRIM38 stabilizes these signaling by promoting SUMOylation of cGAS, protecting it from ubiquitination and degradation, which preserves both cGAS and STING function (63) (**Table 1**). TRIM proteins act as crucial checkpoints in the cGAS-STING pathway. Their dual regulatory roles underscore their significance in innate immune homeostasis and highlight their potential as therapeutic targets in immune-mediated diseases.

These highlight context-dependent regulation as a fundamental property of TRIM proteins. **Table 1** also summarizes and highlights the TRIMs context-dependent regulatory role in the immune system. Individual TRIMs can function as signal amplifiers or terminators depending on the receptor engaged, ubiquitin linkage type, cellular compartment and stage of immune activation. This duality enables fine-tuned immune control but also represents a major challenge for therapeutic targeting, as indiscriminate inhibition or activation may disrupt immune homeostasis.

## 5 TRIM proteins in adaptive immunity

### 5.1 TRIM proteins in T-cell activation

Although TRIM proteins are extensively characterized for their roles in innate immune regulation, they also exert critical and multifaceted functions in adaptive immunity, particularly in the activation of T-cells, their differentiation, and maintaining the state of tolerance. Through ubiquitination-dependent mechanisms, TRIMs regulate essential signaling intermediates, transcriptional regulators, and receptor complexes, establishing a balanced immune activation while preventing autoimmunity. Compared with innate immunity, mechanistic studies of TRIM proteins in adaptive immune cells remain limited, and most available evidence is derived from a small number of well-characterized TRIM family members.

Several TRIM family members act as E3 ubiquitin ligases that conjugate distinct ubiquitin chain linkages. K63-linked chains promote signaling complex formation and K48-linked chains mediate proteasomal degradation at early steps of T-cell receptor (TCR) signaling. Certain TRIMs stabilize TCR complexes by interacting with the TCR ζ-chain, enhancing receptor surface expression and calcium mobilization, thereby enhancing T-cell activation (107). Upon TCR engagement, kinases such as Lck (Src-family) and ZAP-70 (Syk-family) are activated, triggering downstream pathways including PI3K–Akt, MAPK, NF-κB, and AP-1, which collectively promote IL-2 transcription, T-cell proliferation, and survival (107–109). TRIM proteins also play key roles in T-cell lineage differentiation, influencing the balance between effector subsets such as Th2 cells and regulatory T cells (Tregs). Certain TRIMs promote Th2 differentiation associated with allergic inflammation, whereas others negatively regulate Th2-driven immune responses (110). Conversely, specific TRIMs can suppress Treg differentiation in CD4^+^ T cells, thereby predisposing to autoimmune disease development (111). Beyond activation and differentiation, TRIMs modulate peripheral T-cell tolerance by regulating activation thresholds, managing effector functions, and preventing intense proliferation (112).

TRIM27 acts as a negative modulator of TCR signaling by mediating ubiquitin-dependent degradation of proximal signaling molecules and limiting PI3K/Ca²^+^ flux, thereby suppressing CD4^+^ T-cell proliferation and cytokine release (113). TRIM21 (also known as Ro52) is a well-studied TRIM that is involved in innate as well as adaptive immunity, and functions as a modulator of the IL-23/Th17 axis negatively (114). TRIM21 has a role in T-cell activation since the overexpression of TRIM21 in Jurkat cells increases IL-2 synthesis after CD28 stimulation, while TRIM21 knockdown in Jurkat cells show decreased IL-2 induction (115). TRIM21 deficiency in mice results in excessive proliferation of T-cells and increased secretion of inflammatory cytokines such as IL-6 and IL-17, summarizing features of systemic lupus erythematosus through the IL-23-Th17 pathways (116). Mechanistically, TRIM21 may influence the IRF4/IRF5 axis, thereby promoting differentiation of antibody-secreting plasma cells (117).

TRIM30α negatively regulates T-cell signaling by promoting lysosomal degradation of TAK1-binding protein 2 (TAB2) and TAB3, leading to inhibition of the TAK1–NF-κB axis and reduced T-cell activation (57). In mice, TRIM30 deficiency alters CD4/CD8 T-cell ratios, enhances T-cell proliferation in some settings, but can diminish IL-2 production by downstream NF-κB, suggesting a context-dependent role in maintaining homeostatic activation thresholds (118). In contrast, TRIM28 functions as a positive modulator of CD8^+^ T-cell activation through epigenetic mechanisms. It orchestrates chromatin remodeling and three-dimensional chromatin looping at crucial activation-associated genes, such *as Ifng, Gzmb, Tbx21*, and IL2, thereby promoting transcriptional activation during cytotoxic responses (119). TRIM28 also regulates T helper 17 (Th17) and Treg differentiation, influencing the balance between immune tolerance and autoimmunity (120). In human T cells, TRIM28 interacts with Foxp3 through the TRIM28–FIK–Foxp3 complex, which is essential for the suppressive function of Tregs. Disruption of this complex revokes Treg suppressive capacity and leads to dysregulated Foxp3 target gene expression, a mechanism implicated in autoimmune pathogenesis (121).

In signal transducer and activator of transcription 1 (STAT1) signaling, TRIM24 acts as a negative regulator. It interacts with nuclear receptors, including retinoic acid and estrogen receptors, along with chromatin-associated factors, thereby inhibiting STAT1-mediated transcriptional activity (122). TRIM24 expression is elevated in Th2 cells, where it contributes to allergic asthma pathogenesis by dampening IL-1-induced gene expression and mitigating IL-1β-driven bronchial inflammation (123). Furthermore, TRIM32 deficiency enhances Th2 cell differentiation and cytokine production, exacerbating atopic dermatitis, suggesting that TRIM32 functions as a negative regulator of Th2-mediated allergic responses (124).

### 5.2 TRIM proteins and B-cell function

TRIM proteins function predominantly as E3 ubiquitin ligases or scaffold adaptors, modulating B-cell responses at multiple levels. They influence B-cell receptor (BCR) and TLR signaling intermediates through K63 or K48-linked ubiquitination, either enhancing or terminating downstream signaling. Additionally, TRIMs recruit deubiquitinases or autophagy receptors to stabilize or remove signaling complexes and regulate chromatin accessibility and transcription factor activity, establishing long-term functional programs in B cells (73). Through these mechanisms, TRIM proteins exert broad control over adaptive immunity, modification B-cell activation, antigen processing, nucleic acid sensing, and transcriptional programs that dictate activation thresholds, class-switch recombination, and immune tolerance. Beyond direct B-cell effects, TRIMs also modulate T-cell functions and differentiation, contributing to the overall regulation of immune homeostasis, as discussed in the previous section.

Abnormal B-cell differentiation or apoptosis can lead to autoimmune disorders. For example, in a recent study, *Trim21* deletion enhances B-cell proliferation and production of antibodies, primarily due to defects in B-cell rather than effects caused by T cells or DCs. TRIM21 is also a known autoantigen that binds anti-SS-A antibodies, commonly identified in the sera of individuals with autoimmune disorders (46, 117). TRIM proteins further regulate B-cell function via the noncanonical NF-κB pathway. TRIM55 mediates the ubiquitination of p100, facilitating its processing into the transcription factor p52. In mice with B-cell specific loss of *Trim55*, germinal center development and antibody synthesis were markedly reduced, suggesting TRIM55 is a possible therapeutic target in autoimmune disorders (125).

TRIM33 is another key regulator that governs the homeostasis and function of multiple immune cell types, including DCs, which serve as sentinels of the immune system (126). It is found in *Trim33*^fl/fl^ Cre-ER^T2^ mice that TRIM33 deletion impaired DCs differentiation from hematopoietic progenitors across developmental stages. This deficiency also downregulated essential genes such as *Irf8* and *Bcl2l11* for DCs differentiation, maintenance and survival. TRIM33 thereby highlights its role as a critical regulator of both innate and adaptive immunity (127). Available evidence from mechanistic studies in adaptive immunity is limited but indicates that TRIM proteins regulate B-cell biology, shape humoral immunity, maintain immune tolerance, and influence autoimmune disease susceptibility.

## 6 TRIM dysregulation in autoimmune and immune-related disorders

### 6.1 Systemic lupus erythematosus

A classic autoimmune disease, systemic lupus erythematosus (SLE) is characterized by the generation of autoantibodies, dysregulated type I IFN signaling, and multi-organ involvement. A hallmark of SLE is the presence of elevated autoantibodies against TRIM21 (Ro52), which can impair its E3 ligase activity. Another characteristic is the disruption of the negative mediation of type I IFN signaling, and alters the differentiation of B-cell. This contributes to a self-amplifying inflammatory loop that exacerbates disease activity (128). TRIM21 mediates ubiquitination and proteasomal degradation of critical substrates, including STING, thereby restricting type I IFN production. In *Trim21* deficient mice, lupus-like pathology emerges, characterized by expansion of Th17 cells and elevated anti-double-stranded DNA antibodies (129).

Furthermore, TRIM5 has been associated with SLE pathogenesis. In peripheral blood mononuclear cells (PBMCs) from individuals with SLE, it is strongly expressed and correlates with the expression of antiviral genes. Elevated TRIM5 levels are associated with reduced naive CD4^+^ T cells and an increased proportion of M2 macrophages, which modulate immune responses in SLE. TRIM5 can inadvertently activate innate pathways of NF-κB and type I IFN production, thereby amplifying inflammatory responses and contributing to disease progression (130). Inhibition of IFN has become a new treatment for SLE patients and these observations position TRIM21 and TRIM5 as potential therapeutic targets to restore immune homeostasis and limit tissue damage in SLE.

### 6.2 Sjögren’s syndrome

Sjögren’s syndrome (SS) is an autoimmune disease that mostly affects exocrine glands, especially the salivary and lacrimal glands. It causes lymphocytic infiltration and glandular dysfunction. TRIM21/Ro52 and corresponding autoantibodies (anti-TRIM21/Ro52) are well-recognized autoantigens in SS and other autoimmune diseases, including SLE (131). Detection of anti-TRIM21 antibodies is a key diagnostic marker for SS.

Under physiological conditions, TRIM21 inhibits type I IFN signaling by ubiquitin-mediated degradation of IRF3/5. In SS patients, PBMC analyses reveal elevated TRIM21 transcript levels, coupled with persistently high IRF3 and IRF5 protein expression. TRIM21/Ro52 transcription is upregulated by IRF1/2 and suppressed by IRF4/8, consistent with its overexpression in SS. Autoantibodies against TRIM21 impair its ability to degrade IRFs, resulting in dysregulated IFN production and elevated pro-inflammatory cytokines, subsequently high TRIM21/Ro52 expression (132, 133). *In vitro* studies demonstrate that anti-TRIM21 antibodies can sterically hinder the interaction between TRIM21 and its E2 ubiquitin-conjugating enzyme, hence preventing normal autoubiquitination and degradation of substrates (128). This dysregulation amplifies autoimmunity in SS and highlights it as a potential therapeutic target by either restoring TRIM21 function or alleviating the effects of anti-TRIM21 antibodies.

### 6.3 Inflammatory bowel disease and Crohn’s disease

Inflammatory bowel disease (IBD), which includes Crohn’s disease (CD) and ulcerative colitis (UC), is defined by chronic relapsing intestinal inflammation, impaired epithelial barrier function, dysregulated immune responses, and genetic predisposition. TRIM proteins contribute to both susceptibility and progression of IBD through their modulation of immunological signaling and maintenance of gut homeostasis.

One prominent TRIM member associated with the susceptibility of IBD is TRIM20, which is encoded by the MEFV gene (134). TRIM21 suppresses pro-inflammatory cytokines in the gut and inhibits differentiation of CD4^+^ T cells into Th1 and Th17 inflammatory subsets (135). TRIM22 has been identified as a signaling modulator of nucleotide-binding oligomerization domain containing 2 (NOD2), essential for microbial recognition and inflammatory responses. Variants in the SPRY domain of TRIM22 impair its K63-linked ubiquitination of NOD2, consequently weakening NOD2-dependent muramyl dipeptide (MDP) signaling and promoting Crohn’s disease-associated inflammation (136).

TRIM27 increases gut inflammation by promoting the STAT3 and JAK1 interaction, leading to the activation of STAT3 and the amplification of pro-inflammatory signaling in the colon. Knockout studies in mice demonstrate resistance to dextran sulfate sodium (DSS)-induced colitis, supporting the pro-inflammatory role of TRIM27 protein (137). TRIM27 also stabilizes β-catenin, thereby activating Wnt/β-catenin signaling to promote the self-renewal of intestinal stem cells. Knockout of Trim27 impairs organoid formation, a process that can be restored by reintroducing either TRIM27 or β-catenin. This underscores the therapeutic potential of addressing the TRIM27/Wnt/β-catenin pathway in IBD (138). TRIM31 regulates intestinal inflammation by promoting ubiquitination and proteasomal degradation of NLRP3, a critical component of the inflammasome that facilitates IL-1β secretion. By attenuating inflammasome activity, TRIM31 protects against excessive intestinal inflammation (139). Dysregulated TRIM activity in the gut compromises barrier integrity, alters T-cell differentiation, and disrupts the balance between pro-inflammatory and regulatory T-cell subsets, which further drives the inflammatory cascade associated with IBD.

### 6.4 Familial mediterranean fever

Familial Mediterranean fever (FMF) is an autoinflammatory condition marked by repeated episodes of fever, serositis, and systemic inflammation. This disorder is primarily caused by changes in the MEFV gene, which makes the TRIM20 (pyrin). These mutations disrupt TRIM20-mediated ubiquitination and the regulated degradation of inflammatory mediators, resulting in persistent activation of inflammatory-related pathways and increased release of IL-1β, a central hallmark of FMF pathogenesis (140). The sustained IL-1β signaling drives systemic inflammatory responses and, in some cases, contributes to intestinal inflammation resembling IBD.

### 6.5 Psoriasis

Psoriasis is a persistent inflammatory dermatological condition marked by keratinocyte proliferation, aberrant immune activation, and the formation of scaly skin plaques. Multiple TRIM proteins play essential roles in modulating inflammatory responses within keratinocytes.

Increased expression of TRIM21 has been noted in psoriatic epidermal cells, and its suppression in human keratinocytes significantly reduces the synthesis of inflammatory cytokines and chemokines (141). The E2 ubiquitin-conjugating enzyme UBE2L3 regulates TRIM21 activity, and its overexpression decreases STAT3 pathway activation and IL-1β levels. In a mouse model of psoriasis induced by imiquimod (IMQ), reduced UBE2L3 expression was associated with caspase-1 activation in the epidermis. Overexpression of UBE2L3, in turn improved psoriasis-like lesions and reduced levels of both pro-IL-1β and mature IL-1β (142). Similarly, silencing of TRIM21 decreased the release of TNF-α, IL-6, and IL-1β in cells stimulated by LPS (143). Furthermore, TRIM33 is markedly upregulated in psoriatic lesions and acts as a positive modulator of inflammation in keratinocytes. TRIM33 promotes the ubiquitination of Annexin A2 (Anxa2), hence promoting its interaction with NF-κB subunits p50 and p65, which enhances transcription of downstream pro-inflammatory genes such as IL-6, IL-1β, and NLRP3 (144). The elevated epidermal expression of TRIM33 in psoriasis underscores its contribution to disease pathology, emphasizing the therapeutic potential of targeting TRIM-mediated signaling pathways to mitigate psoriatic inflammation.

### 6.6 Type 1 diabetes

Type 1 diabetes (T1D) is an autoimmune disorder in which the immune system destroys pancreatic β-cells. Both genetic and functional evidence suggest that TRIM27 mutations correlate with increased susceptibility to T1D. TRIM27 exerts a protective role in pancreatic tissue by suppressing TNF-α-induced β-cell death. In Trim27-deficient mice, exposure to streptozotocin (STZ) induces severe diabetes, reflecting enhanced β-cell apoptosis. Mechanistically, TRIM27 regulates the deubiquitination of receptor-interacting protein 1 (RIP1), preventing caspase-3 activation and β-cell apoptosis (**Table 2**). Loss of TRIM27 leads to decreased β-cell mass and an increase of cytokines such as IFN-γ and IL-1β (153). TRIM72 is known as Mitsugumin 53 (MG53), functions as an E3 ligase that modulates insulin signaling and membrane repair in β-cells. Dysregulation of TRIM72 leads to insulin resistance and β-cell dysfunction through ubiquitin-mediated degradation of the insulin receptor and insulin receptor substrate-1 (IRS-1), thereby disrupting glucose and lipid metabolism (154, 155). Further functions of the TRIM72 protein in insulin signaling and its potential as a therapeutic target in human insulin resistance and type 1 diabetes require more research.

**Table 2:** Reported mechanistic evidence for TRIM proteins in autoimmune diseases.

Autoimmune disease	TRIM proteins	Mechanistic evidence
SLE	TRIM21(Ro52), TRIM5, TRIM68	TRIM21 autoantigen; regulates IFN pathway and T-cell activation (39, 128). TRIM5 & TRIM68 linked via expression and autoantibody reactivity (130, 145).
SS	TRIM21(Ro52), TRIM68	TRIM21 is a clinical autoantigen, associated with disease severity (128, 131). TRIM68 identified as autoantigen (145).
RA	TRIM38, TRIM3, TRIM72, TRIM33	TRIM38 regulates TRAF6/NF-κB signaling (146). TRIM3 & TRIM72 participate in synoviocyte function (147, 148). TRIM33 affects inflammation and autoantibody responses (149).
MS	TRIM21, TRIM30α, TRIM32	TRIM21 & TRIM32 involved in neuroprotection and anti-inflammatory responses (150, 151). TRIM30α modulates TLR/NF-κB signaling (103).
Psoriasis	TRIM21, TRIM33	TRIM21 associations with skin autoantibodies and inflammation (142, 143). TRIM33 enhances keratinocyte inflammation (144).
IBD/CD	TRIM22, TRIM21, TRIM28	TRIM22 regulates NOD2 signaling (136). TRIM21 influences Th1/Th17 cell differentiation (135).TRIM28 mediates epigenetic regulation (152).
FMF	TRIM20 (MEFV/Pyrin),	TRIM20 (Pyrin) is the classical gene, regulating inflammasome and IL-1 signaling by interacting with NALP3 and caspase-1 (140).
T1D	TRIM27, TRIM72	TRIM27 gene variants associated with β-cell immunity (153). TRIM72 mediates IRS‐1 degradation and inhibit the constrain signaling (154, 155).

### 6.7 Multiple sclerosis

It is a chronic inflammatory disorder of the central nervous system (CNS) characterized by demyelination, neuroinflammation, and persistent neurological impairment (156). In an experimental autoimmune encephalomyelitis (EAE) model, TRIM21 was found to interact with pyruvate kinase M2 (PKM2), facilitating its nuclear translocation and promoting phosphorylation of STAT3 and NF-κB, as well as interaction with c-Myc. This process enhances astrocyte glycolysis and proliferation, contributing to neuroinflammation in multiple sclerosis (MS) (150). Targeting the TRIM2-PKM2 axis may provide a promising therapeutic approach for MS. Additionally, mutations in TRIM32 have been associated with neuronal damage and demyelination, as reported in a clinical case by Marchuk et al. (2021) (151). Human endogenous retroviruses (HERVs) have been implicated in the pathogenesis of MS by triggering immune and inflammatory responses and disrupting neuronal signaling (157, 158). TRIM proteins such as TRIM5α and TRIM22 regulate antiviral immunity by recognizing retroviral capsids and shaping immune responses to HERVs. Aberrant activation of these pathways can lead to sustained type I IFN and NF-κB signaling and promote the activation of autoreactive T and B cells. This process linking antiviral immune dysregulation drives loss of self-tolerance and contributes to demyelination and neurodegeneration. Aberrant regulation of these TRIM proteins may contribute to immune activation and disease progression in MS (158).

### 6.8 Rheumatoid arthritis

Rheumatoid arthritis (RA) is a chronic autoimmune disorder that primarily affects synovial joints, leading to persistent inflammation, synovial hyperplasia, and cartilage destruction. Fibroblast-like synoviocytes (FLS) play a crucial role in the pathogenesis of RA by promoting the production of TNF-α and IL-1β, thereby propagating inflammation (159).

The TRIM38-NLRP6 axis (nucleotide oligomerization domain-like receptor family pyrin domain containing 6) has been found to negatively affect the canonical NF-κB pathway in RA-FLS through an inflammasome-independent mechanism. TRIM38 interacts with NLRP6 and TAB2/3, inducing their lysosome-dependent degradation. This inhibits NF-κB activation and decreases cytokine production in FLS (146). A recent study has also linked ferroptosis, an iron-dependent form of programmed cell death driven by lipid peroxidation, to the pathogenesis of RA. Inducing ferroptosis in RA-FLS can alleviate disease progression (160).TRIM16 expression is downregulated in RA, while its overexpression suppresses disease severity in mouse models. TRIM16 promotes the ubiquitination and degradation of the transcription factor Snail family zinc finger 1 (Snai1), which normally inhibits ferroptosis. The TRIM16-Snai1 axis enhances ferroptosis and exerts anti-inflammatory effects by counteracting TNF-α, identifying a novel immunoregulatory mechanism and therapeutic target to control prognosis in RA (161).

Additionally, TRIM3 has been shown to suppress FLS proliferation and reduce inflammatory cytokine secretion via the p38 MAPK signaling pathway. Low TRIM3 expression is observed in RA synovial tissue relative to healthy controls, while its overexpression increases p53 and p21expression, leading to cell cycle arrest and decreased production of TNF-α and IL-6 (147). TRIM33 is also downregulated in RA synovial fibroblasts (RASFs). KLF9 is a transcription factor related to proliferation, apoptosis and is widely involved in B and T-cell differentiation. The transcription factor KLF9 positively modulates the expression of TRIM33, which in turn suppresses TNF-α-induced proliferation of RASF and the release of inflammatory cytokines such as IL-1β, IL-6, IL-8. Activation of the KLF9-TRIM33 axis thus restrains abnormal fibroblast proliferation and inflammatory responses, highlighting its potential as a therapeutic target that can intervene and reverse RA progression (149).

## 7 Considerable strategies for TRIM proteins as therapeutic targets

The therapeutic targeting of TRIM proteins involves several interconnected strategies. A detailed understanding of both the molecular structure and functional dynamics of TRIM proteins has enabled the development of innovative approaches to modulate their activity.

### 7.1 Pharmacological modulation strategies

TRIM proteins contain diverse functional domains that enable them to regulate multiple cellular processes, with the RING domain serving as a key determinant of E3 ubiquitin ligase activity. Although ubiquitination represents a central mechanism of action, several TRIM family members also exert ubiquitin-independent functions (15). Effective therapeutic targeting cannot rely solely on direct inhibition of catalytic activity. Instead, successful modulation of TRIM proteins requires a detailed understanding of their multi-domain architecture and adapter-like properties to achieve selective and context-appropriate intervention. These features highlight the need for alternative therapeutic strategies that extend beyond catalytic inhibition, including targeted protein degradation and domain-selective modulation.

**Figure 4:**
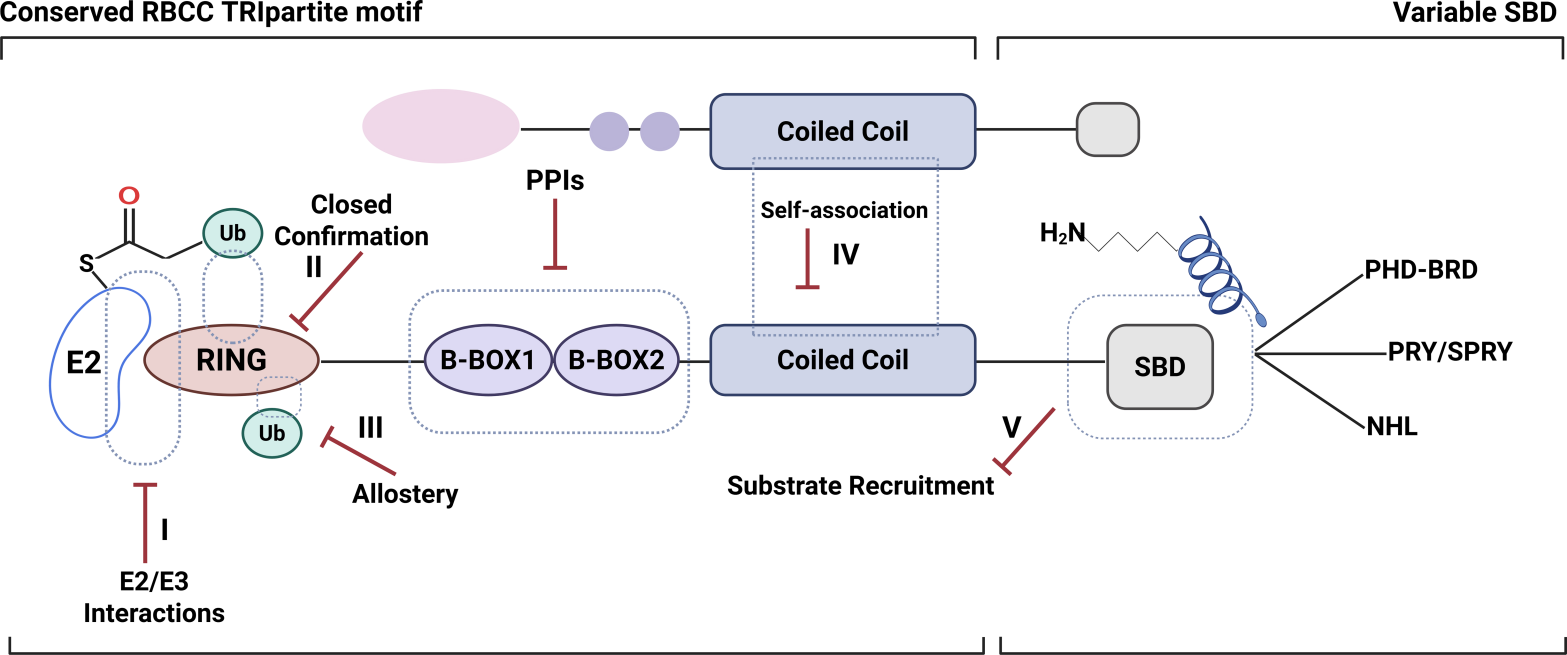
Schematic demonstration of the structural basis and potential targeting options for Tripartite motif (TRIM) proteins.

Therapeutic modulation of TRIM proteins can be broadly divided into direct and indirect approaches. Direct strategies aim to alter the catalytic or scaffolding functions of TRIM by targeting its RING-type E3 ubiquitin ligase domain or substrate-binding (PRY/SPRY) domains (162, 163). The shallow and extended protein-protein interaction (PPI) interfaces of TRIMs complicate conventional small-molecule inhibition. Recent structure-guided designs and fragment-based screening methods have begun to identify druggable allosteric sites. Complementary indirect strategies exploit the broader ubiquitin-proteasome and deubiquitinase networks that regulate TRIM turnover or substrate fate. Inhibitors of deubiquitinases and E2-E3 interface modulators can fine-tune TRIM activity without directly blocking the ligase function (**Figure 4**) (164, 165).

Proteolysis-targeting chimeras (PROTACs) and molecular glues (MGs) are being developed to selectively degrade or stabilize pathogenic TRIM variants. These approaches offer a precision strategy for autoimmune and interferon-driven diseases (166–168). Parallel research efforts also focus on targeting downstream pathways influenced by TRIM proteins, such as cGAS-STING, NF-κB, or TBK1-IRF3. Small-molecule agonists or antagonists may restore balance in dysregulated immune signaling (169, 170). Although no therapeutic drugs currently exist that directly target TRIM proteins at the experimental or clinical stage, their extensive involvement in immunological regulation makes them promising candidates for future drug development. These emerging pharmacological frameworks enable pathway-specific modulation of immune responses and position TRIM proteins as tractable nodes within the ubiquitin landscape, despite historical challenges in targeting E3 ligases.

### 7.2 TRIM-based biomarkers for diagnosis and prognosis

TRIM proteins are increasingly recognized as diagnostic and prognostic biomarkers across autoimmune-related diseases. Among them, anti-Ro52/TRIM21 autoantibodies are well-established serological markers in systemic autoimmune disorders such as SLE and Sjögren’s syndrome. Their expression levels are correlated with disease severity, IFN signatures, and distinct clinical phenotypes (171, 172). The structural domains of TRIM proteins, such as the C-terminal substrate-binding domains (PRY/SPRY, PHD-BRD, NHL), are essential for their molecular interactions or functions and may be exploited for diagnostic targeting (166, 173, 174).

Beyond autoantibodies, altered transcriptional and protein expression of several TRIMs, including TRIM19, TRIM21, TRIM24, TRIM28, and TRIM33, has been associated with disease progression, treatment response, and oncogenic transformation. This demonstrates their effectiveness as molecular classifiers and outcome predictors. The integration of quantitative TRIMs expression assays with high-throughput genomic and proteomic platforms facilitates patient stratification based on immune activation states or TRIMs pathway activity, aiding personalized therapeutic decisions (175–177). Despite their potential, the clinical translation of TRIM proteins as biomarkers remains in its early stages. Large multicenter validation studies are still required to confirm their diagnostic and prognostic significance and to establish standardized assay thresholds for routine clinical use.

### 7.3 Gene therapy, small molecules, and targeted degradation

Recent advancements in gene and protein-level modulation technologies have opened new therapeutic avenues for targeting TRIM proteins. Gene therapy approaches such as RNA interference (RNAi), antisense oligonucleotides (ASOs), and CRISPR-based regulation offer the potential to silence pathogenic TRIM isoforms or restore deficient TRIM expression in a cell type-specific manner, thereby reestablishing immune homeostasis. In parallel, strategically designed small molecules that modulate TRIM E3 ligase or substrate-binding activities are being complemented by breakthroughs in targeted protein degradation technologies, particularly PROTACs and MGs (178–181). These strategies utilize the cellular ubiquitin system to selectively degrade aberrant TRIMs or their downstream effectors. Proof-of-concept studies, such as TRIM24-directed PROTACs, have shown tumor suppression in preclinical cancer models, validating the feasibility of pharmacologically manipulating TRIM protein stability (182). PROTAC technologies can be designed to engage different E3 ligases, enabling the selective degradation of disease-associated or “undruggable” proteins (183, 184). The integration of gene editing, chemical biology, and proteomics-guided screening is producing a comprehensive therapeutic toolkit for modulating TRIM protein activity in immune-related diseases. Delivery specificity and off-target effects continue to pose substantial translational challenges.

### 7.4 Challenges and translational barriers

A major translational barrier in targeting TRIM proteins is their high structural conservation, particularly within the RING domain, which complicates selective inhibition and increases the risk of off-target effects across the TRIM family. Many TRIMs share identical catalytic architectures, making it difficult to achieve specificity using conventional small-molecule inhibitors. In addition, TRIM proteins often exhibit stimulus and cell-type-specific dual functions, acting as either pro-positive or anti-negative regulators of immune signaling. This context-dependent functional plasticity raises the risk that indiscriminate inhibition may disrupt immune homeostasis and exacerbate disease.

Despite encouraging progress, various challenges persist in obstructing the translation of TRIM-targeted therapeutics. Many TRIM proteins do not possess clearly defined ligandable sites, and their complex protein-protein interaction interfaces complicate pharmacological targeting. Their opposing functions in distinct cellular contexts further complicate therapeutic intervention, as targeting a TRIM protein may yield opposing outcomes depending on disease stage, tissue, or immune context. Emerging strategies such as PROTACs and MGs offer opportunities to overcome these challenges by enabling domain-specific targeting and conditional degradation of pathogenic TRIM activities while preserving physiological functions.In this context, PROTAC-based strategies may offer a route to enhanced selectivity by exploiting tissue-based E3 ligase recruiters, engaging TRIM-specific ligandable features outside the highly conserved RING domain, and limiting systemic exposure through localized or targeted delivery approaches such as nanoparticle formulations.

Specificity and safety remain major concerns, as systemic modulation of ubiquitin signaling could interfere with proteostasis and trigger unintended immune responses. Pharmacokinetic and delivery issues, including the stability and bioavailability of PROTACs or small-molecule inhibitors, also present key challenges for *in vivo* applications. Furthermore, species-specific differences in TRIM repertoires and incomplete validation of long-term safety hinder the extrapolation of preclinical findings to humans (22, 185, 186). The lack of selective chemical probes, ligand maps, and validated E3 ligase recruiters further limits experimental precision.

Robust biomarker qualification and patient stratification frameworks are essential for identifying individuals who may benefit from TRIM protein activation versus inhibition strategies. Addressing these gaps through integrated efforts in structural biology, probe development, and translational immunology will be essential to fully realize the therapeutic potential of TRIM proteins in autoimmune and immune-mediated disorders.

## 8 Conclusions

This review summarizes multiple studies highlighting the diverse roles of TRIM family proteins in various immunological disorders, presenting potential pathways for targeted therapies and improving the management of autoimmune diseases associated with TRIM protein dysregulation. The emergence and progression of immune responses are closely entangled with the multifaceted functions of TRIM proteins, which act as key regulatory molecules in both innate and adaptive immunity (**Table 2**) (6, 7, 13). These proteins influence the activity of PRR signaling pathways, modulate the expression of downstream cytokines, and orchestrate complex immune networks that determine the magnitude and nature of immune activation. Importantly, TRIM-mediated regulation of PRR signaling is stimulus-dependent and cell type-specific, underscoring their dynamic and context-dependent roles in immune modulation (26, 71, 109). Many TRIM proteins act cooperatively or in a coordinated manner to regulate immune homeostasis, highlighting their dual potential to either activate or suppress immune responses. This balance is essential for preventing excessive inflammation and maintaining tolerance, thereby reducing the risk of autoimmune pathology.

Despite growing evidence of their immunoregulatory importance, the rational design of TRIM-targeted inhibitors or modulators remains in its beginnings. Approximately one-third of TRIM genes generate alternatively spliced transcripts that encode variants lacking conserved domains. This complexity has limited the functional roles of individual TRIM isoforms and remains poorly explored for most TRIM family members. TRIM19 has seven reported isoforms that display distinct functions. Among these, only TRIM19 isoform 4 enhances IRF3 stability and signaling by promoting proteasomal degradation of the negative regulator PIN1 (13, 38).The lack of detailed structural and functional characterization, combined with the absence of known active compounds and the inherent multidomain, multifunctional nature of TRIM proteins, presents significant challenges for drug discovery. The review also outlined emerging therapeutic strategies for targeting TRIM proteins, emphasizing their potential as novel molecular nodes for precision immunomodulation. However, species-specific variations continue to complicate preclinical validation and translational extrapolation to human systems. Moving forward, comprehensive investigations are required to deepen our understanding of TRIM biology and identify efficient, isoform-specific targeting strategies. Future research should define isoform-dependent mechanisms, interaction networks, and post-translational modifications that govern the functions of TRIM proteins across distinct immune cell subsets. Advancements in structural biology, proteomics, and single-cell transcriptomics will be instrumental in uncovering actionable molecular targets and guiding the development of next-generation therapies. Such progress will not only enhance our understanding of TRIM-mediated immune regulation but also pave the way for precision therapeutic interventions in autoimmune and immune-mediated diseases.
